# Incidence and prevalence of type 2 diabetes by occupation: results from all Swedish employees

**DOI:** 10.1007/s00125-019-04997-5

**Published:** 2019-09-17

**Authors:** Sofia Carlsson, Tomas Andersson, Mats Talbäck, Maria Feychting

**Affiliations:** 1 Institute of Environmental Medicine, Karolinska Institutet, SE-171 77 Stockholm, Sweden; 2 Centre for Occupational and Environmental Medicine, Stockholm County Council, Stockholm, Sweden

**Keywords:** Epidemiology, Incidence, Lifestyle, Occupation, Prevalence, Prevention, Registry-based, Type 2 diabetes

## Abstract

**Aims/hypothesis:**

The workplace is a potentially important arena for prevention of type 2 diabetes and the first step is to identify occupations where the disease is common and/or risk is high. Therefore, our aim was to analyse incidence and prevalence of type 2 diabetes across all occupational groups in Sweden.

**Methods:**

This nationwide study included all Swedish citizens born between 1937 and 1979 and gainfully employed between 2001 and 2013 (*N* = 4,550,892), and followed for a diagnosis of diabetes from 2006 to 2015 (*n* = 201,717) through national registers. Prevalence in 2013 (mean age 51 years; range 35–67) and age-standardised incidence (per 1000 person-years) were analysed across the 30 most common occupations among men and women. Information on BMI, physical fitness and smoking was obtained through the National Conscription (mean age 18) and Medical Birth Registers (mean age 29).

**Results:**

Prevalence of type 2 diabetes was 5.2% in men and 3.2% in women; in men it was highest among motor vehicle drivers (8.8%) and in women it was highest among manufacturing workers (6.4%). Incidence varied dramatically across occupational groups. In men, it was highest among manufacturing workers (9.41) and professional drivers (9.32) and lowest among university teachers (3.44). In women, incidence was highest in manufacturing workers (7.20) and cleaners (6.18) and lowest in physiotherapists (2.20). We found major differences in the prevalence of being overweight and smoking and in the level of physical fitness across these occupational groups even at young ages.

**Conclusions/interpretation:**

Professional drivers, manufacturing workers and cleaners have a threefold increased risk of type 2 diabetes compared with university teachers and physiotherapists. These differences most likely reflect dramatic differences in the prevalence of lifestyle risk factors. If workplace interventions could reduce weight and increase physical activity among employees in these occupations, major health gains may be made.

**Electronic supplementary material:**

The online version of this article (10.1007/s00125-019-04997-5) contains peer-reviewed but unedited supplementary material, which is available to authorised users.

## Introduction

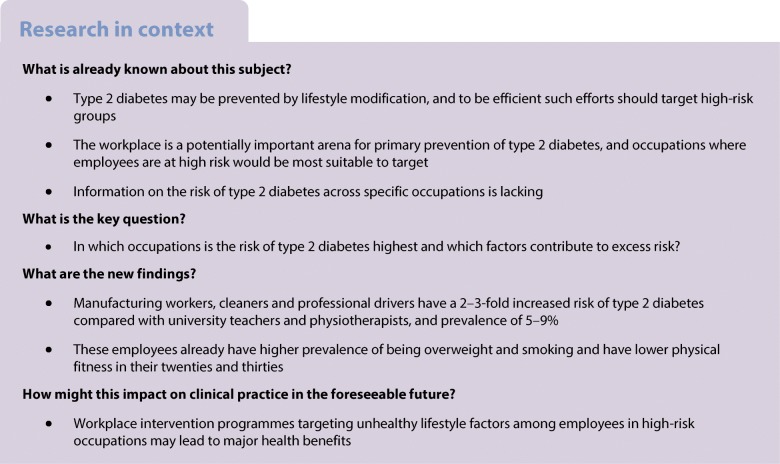



Diabetes is a common and increasing public health problem; according to the International Diabetes Federation, 425 million adults had diabetes in 2017 [1], the global number of afflicted individuals has quadrupled since the 1980s [2] and this trend is expected to continue [1, 3, 4]. For example, in Sweden, prevalence is projected to rise from 7% in 2013 to 10% by 2050 [4]. Demographic changes contribute to the rise but lifestyle changes are also important [2]. Type 2 diabetes is the most common form of diabetes (>85% cases) and risk factors include obesity, physical inactivity, unhealthy dietary patterns and smoking [5]. To reduce the diabetes burden, we need efficient preventive programmes targeting these factors and, to be cost effective, we should focus on high-risk individuals.

The risk of diabetes is known to differ by socioeconomic status (SES). According to a meta-analysis, a 30–40% increased incidence is observed in those with low socioeconomic position as indicated by income, education or occupation [6]. Individuals with low SES form a large and heterogeneous group that may encompass occupational groups with major differences in diabetes risk as well as individuals who are unemployed. A high risk could be due to a high prevalence of obesity, smoking or lack of leisure time physical activity among the employees. In addition, some occupations may promote diabetes; for example, those that involve shift work [7], long sitting times [8] or psychological stress [9]. Adults spend a large proportion of their time at work, which means that the work place could be an arena for primary prevention, and the first step towards such an approach would be to identify occupations where the diabetes risk is high. Diabetes prevalence is also important to map, since major gains in productivity could potentially be made if efficient secondary prevention could reduce the risk of comorbidities in high-prevalence occupations.

To date, few studies have investigated the diabetes burden across different occupational groups [10, 11]. To fill this knowledge gap, we investigated the prevalence and incidence of type 2 diabetes by occupation, using nationwide, register-based data for the entire Swedish population.

## Methods

### Study population

**Fig. 1:**
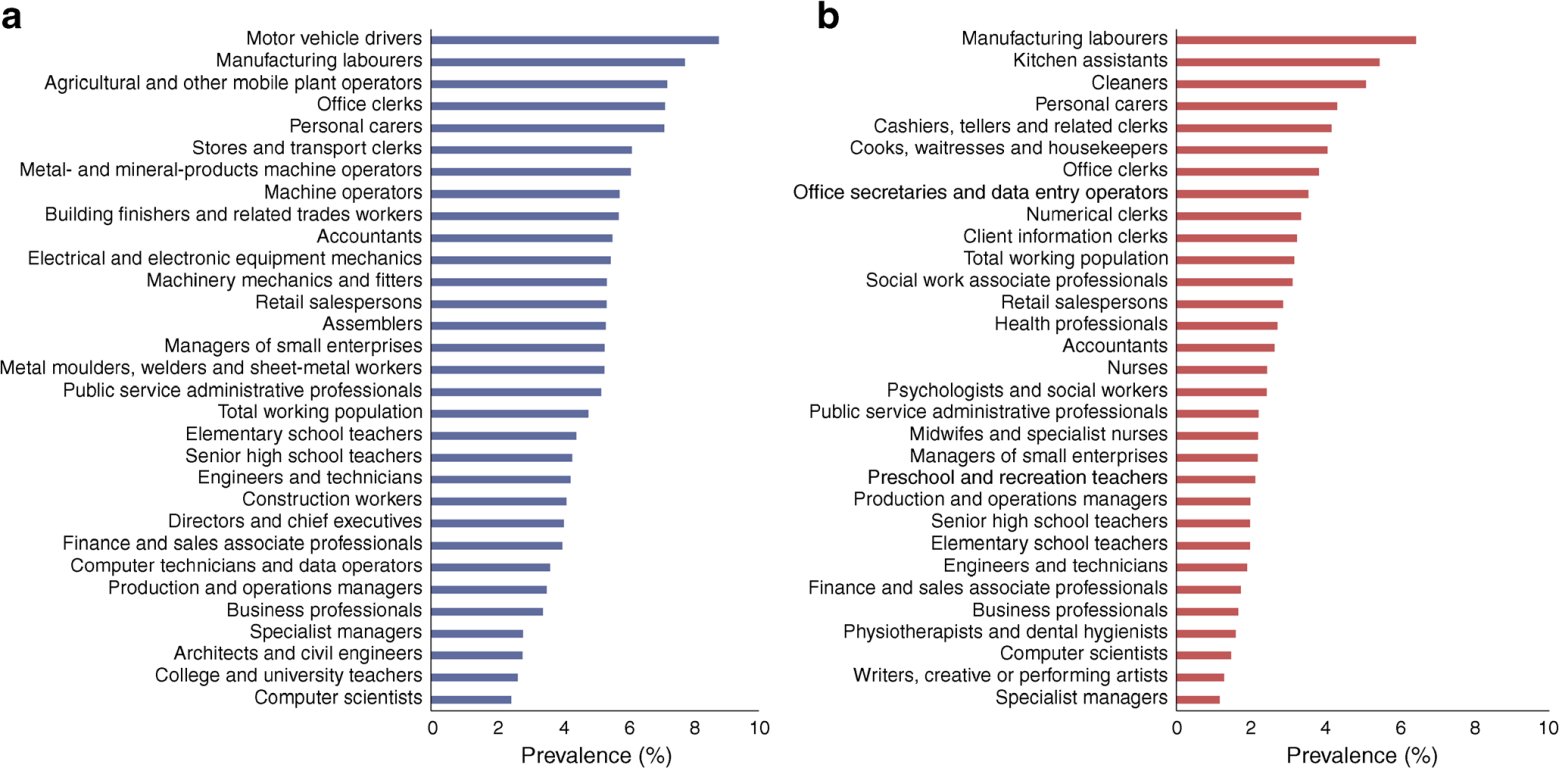
Prevalence (%) of type 2 diabetes across the 30 most common occupations in men (**a**) and women (**b**) in Sweden in 2013

This nationwide study is based on linkage between the National Population, Prescription, Patient, Cause of Death, Medical Birth and Military Conscription Registers and LISA (Longitudinal Integrated Database for Health Insurance and Labour Market Studies) [12] (Electronic Supplementary Material [ESM] Fig. 1). From the Swedish Total Population Register [13] we identified all Swedish citizens who were born between 1937 and 1979 (*N* = 5,445,478). Of these, 4,550,892 (83.6% overall; 83.2% of all men and 84.7% of all women) were gainfully employed for at least 2 years between 2001 and 2013 and constituted our study population. The birth years of the study population were chosen to ensure that the person was less than 65 years old in 2001 (in order to have information on occupation) and old enough to turn 35 before end of follow-up in 2015. These individuals were followed for incidence of diabetes from 2006 to 2015. We chose 2006 as the first year of follow-up since the National Prescription Register was introduced in 2005 and this was our primary source of diabetes information (described below). It was an open cohort study where people were followed from 2006 or from the year they turned 35 if they were younger than 35 in 2006. We used the unique personal identification number assigned to all Swedish residents to link these individuals to other Swedish nationwide registries.

### Occupation and covariates

Information on occupation and education for the period 2001–2013 was obtained by linkage to the LISA registries; LISA is a longitudinal database for health insurance and labour market studies that integrates data from the educational and labour market sectors [12]. LISA holds annual registers starting from 1990 and includes all Swedish citizens, ≥16 years of age as of December 31, each year. Data on main occupation are recorded in November of each year based on the occupation with the highest taxable salary [12]. This information is classified according to the Swedish Standard Classification of Occupations 1996 (SSYK96) [14], which is a national version of the International Standard Classification of Occupations [15]. Military conscription data on physical fitness and BMI were available for men who enlisted between 1969 and 1999 (57% of the male study population). The Military Conscription Register holds data from the 2 day conscription assessment that was mandatory for all Swedish men until 2009. The majority attended the conscription at age 18 and more than 95% of all Swedish men completed the assessment. Weight and height were recorded and used to calculate BMI (kg/m^2^). Physical fitness was measured using cycle ergometer testing which assessed maximal aerobic workload (*W*_max_). In women, we used information from the Medical Birth Register which records data on all women who gave birth from 1973 onwards. This register has information on smoking, height and weight recorded in the first trimester which we used to calculate BMI. Of the 2,226,049 women in the study, 50.5% had given birth from 1973 onwards and therefore had information recorded in the Medical Birth Register. If a woman had information from several births recorded, we used the first recording.

### Diabetes

We identified individuals with diabetes by record linkage to the National Patient and Prescription Registers. The Patient Register contains nationwide information on all diagnoses from hospital admissions since 1987 and outpatient specialist care since 2001, coded according to the Swedish version of the International Classification of Disease (ICD-10 since 1997; www.who.int/classifications/icd/en/) [16]. The Prescribed Drug Register records all filled prescriptions since July of 2005, according to the Anatomical Therapeutic Chemical Classification System [17]. Anatomical Therapeutic Chemical group A10 (insulin and oral glucose-lowering drugs) was used to identify diabetes. In the Patient Register, ICD codes E11 (type 2 diabetes) and E14 (unspecified diabetes) were used as indicators of type 2 diabetes. If the case was identified through the Prescription Register, age at first prescription ≥35 years was used as an indicator of type 2 diabetes. Incident cases that occurred from 2006 to 2015 were identified through these national registries and dated according to the first available record. Individuals with diabetes (any type) recorded prior to baseline (2006 or before entering the cohort at age 35) in the Prescription or Patient Registers were excluded from the incidence analyses. Prevalent cases in 2013 were identified through the same registries.

### Statistical analyses

At the three-digit level there are 113 occupational groups in SSYK96 (there are 355 occupations at the four-digit level, but we deemed this categorisation to be too specific for this report) [14]. In the main analyses, we chose to focus on the 30 most common occupations in men and women (based on total person-time in each occupation), but incidence and prevalence of type 2 diabetes across the full range of occupations are given in ESM Table 5. To be classified into a specific occupational group, a person was required to be in the same occupation for two consecutive years. We calculated sex- and age-standardised incidence of type 2 diabetes from 2006 to 2015 and 95% CIs across occupational groups using the age and sex distributions of the total working population from 2006 to 2015 as weights. Follow-up ended on 31 December 2015, or at the date of recording of diabetes, death or emigration, whichever occurred first. Standardised incidence rate ratios (SIRs) were also calculated for each occupation compared with the total working population. We also estimated population attributable risk percentage for the 30 most common occupations by subtracting the incidence in the occupation with the lowest incidence from the incidence in each specific occupation, then multiplying the difference by the person-years in that occupation and dividing by the number of cases in the total working population. This number shows the proportion of cases that would be eliminated if the incidence in a specific occupation was similar to that of the low-risk occupation. Prevalence of type 2 diabetes in 2013 (the last year for which we had information on occupation) and 95% CIs were also calculated by occupation, and these estimates were not age standardised since we wanted them to reflect the actual diabetes burden across different occupations. As a supplement, age-standardised (using the age distribution of the total working population) prevalences in men and women are given in ESM Tables 1 and 2. In subanalyses, type 2 diabetes prevalence was estimated separately in men and women aged ≥55 years, since prevalence among older employees is interesting to consider in relation to retirement age. We also estimated the age-standardised incidence of type 2 diabetes for the period 2006–2015 in individuals who did not have an occupation recorded in LISA for two consecutive years between 2001 and 2013, and prevalence in 2013 for individuals without an occupation recorded in 2013.

## Results

### Characteristics

Of the study subjects, 85.3% were born in Sweden, 9.1% in other European countries and 5.7% outside of Europe (Table 1). With regard to the highest level of education, 18.7% of men and 14.0% of women had primary school education and 33.4% (men) vs 40.1% (women) had a university education. In 2013, there were 150,131 prevalent cases of type 2 diabetes, and, during follow-up from 2006 to 2015, 201,717 incident cases of type 2 diabetes were identified over 38,838,616 person-years.

**Table 1:** Characteristics of the study population: all Swedish citizens born between 1937 and 1979 who were gainfully employed between 2001 and 2013

Characteristic	All	Men	Women
Total no.	4,550,892	2,295,390	2,255,502
Age at baseline (years), *n* (%)			
35–44	2,070,039 (45.5)	1,059,258 (46.1)	1,010,781 (44.8)
45–54	1,059,412 (23.3)	529,683 (23.1)	529,729 (23.5)
≥ 55	1,421,441 (31.2)	706,449 (30.8)	714,992 (31.7)
Education, *n* (%)			
Primary school	740,283 (16.3)	426,085 (18.7)	314,198 (14.0)
Secondary school	2,126,992 (47.0)	1,093,448 (47.9)	1,033,544 (45.9)
University	1,662,955 (36.7)	761,105 (33.4)	901,850 (40.1)
Country of origin, *n* (%)			
Born in Sweden	3,880,827 (85.3)	1,961,628 (85.5)	1,919,199 (85.1)
Born in Europe outside Sweden	412,849 (9.1)	200,649 (8.7)	212,200 (9.4)
Born outside Europe	257,216 (5.7)	133,113 (5.8)	124,103 (5.5)
Type 2 diabetes			
No. prevalent cases in 2013	150,131	93,997	56,134
Prevalence per 100 in 2013 (95% CI)	4.19 (4.17, 4.21)	5.19 (5.15, 5.22)	3.17 (3.14, 3.20)
No. incident cases 2006–2015	201,717	123,114	78,603
Incidence per 1000 person-years (95% CI)	5.19 (5.17, 5.22)	6.36 (6.33, 6.40)	4.03 (4.00, 4.06)

### Prevalence of type 2 diabetes by occupation

Overall, 4.2% of the Swedish working population had diabetes in 2013, with prevalence higher in men (5.2%) than in women (3.2%). Prevalence (crude) was above 7% in male motor vehicle drivers and manufacturing labourers, whereas only 2.5% of male computer scientists had diabetes (Fig. 1 and ESM Table 1). In women, prevalence (crude) was highest in manufacturing workers (6.4%), cleaners (5.1%) and kitchen assistants (5.5%) and lowest among specialist managers (1.2%) (Fig. 1 and ESM Table 2). Age standardisation had only minor influence on the prevalences (ESM Tables 1 and 2). Separate analyses in individuals 55 years or older (ESM Tables 3 and 4) show that, in men, prevalence of diabetes is 14.9% in manufacturing workers and 14.2% in motor vehicle drivers. In women aged ≥55 years, the highest prevalence is seen in manufacturing workers (10.7%), cleaners (8.3%) and kitchen assistants (8.7%). Prevalence of type 2 diabetes in individuals without a recorded occupation in 2013 was 8.9% overall, 9.7% in men and 8.2% in women.

### Incidence of type 2 diabetes by occupation

**Fig. 2:**
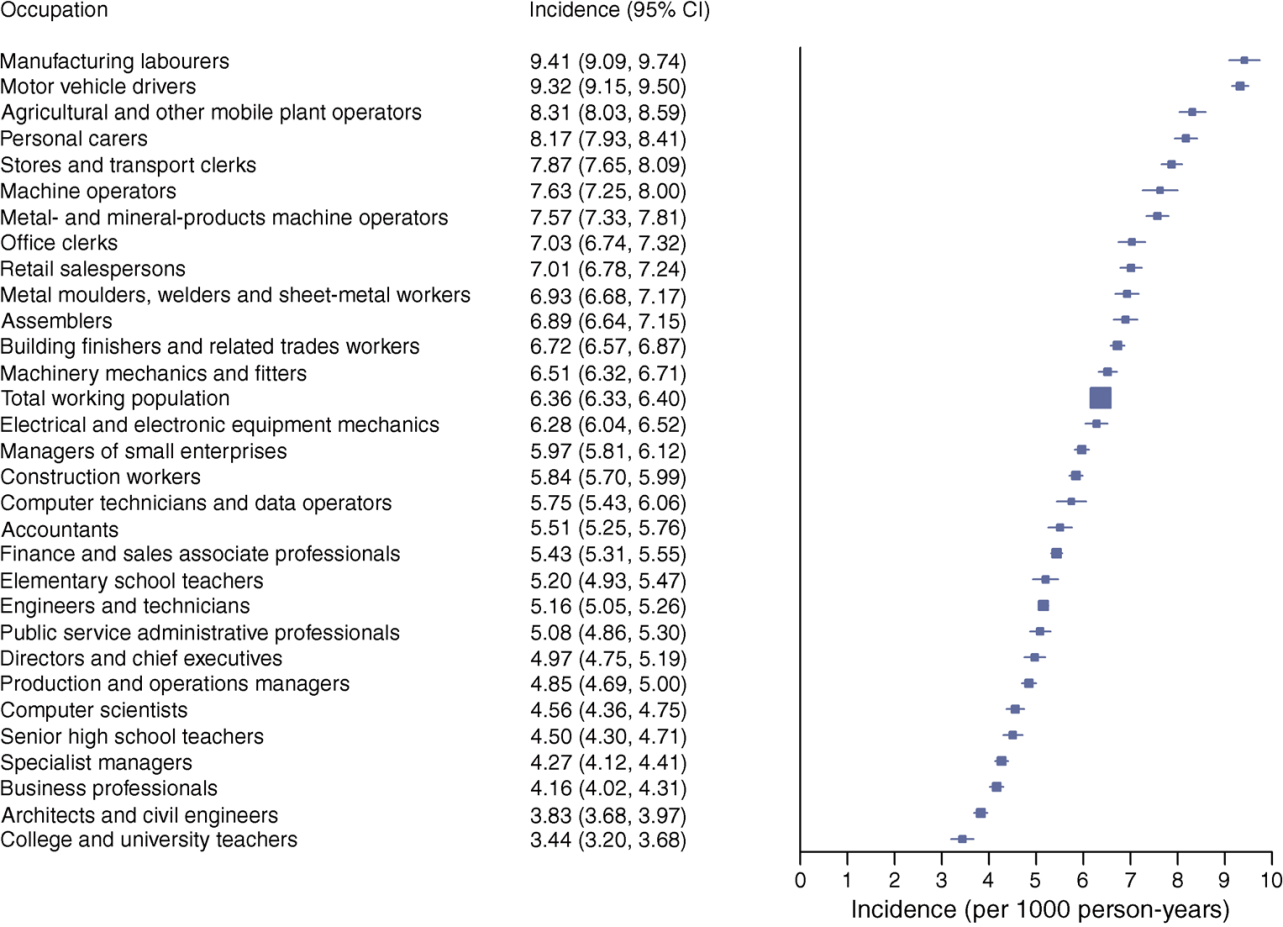
Age-standardised incidence (per 1000 person-years) of type 2 diabetes from 2006 to 2015 across the 30 most common occupations in men in Sweden

**Fig. 3:**
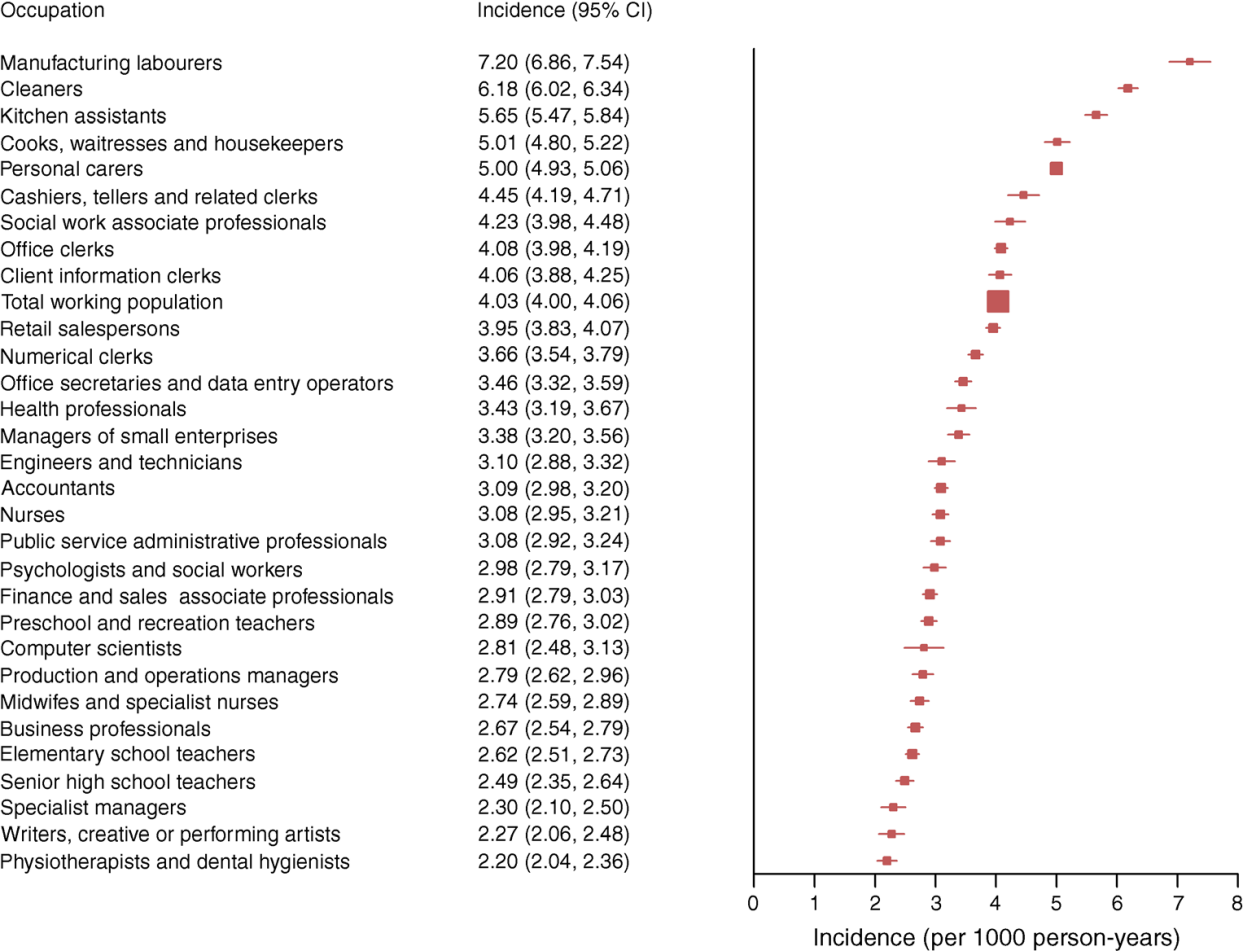
Age-standardised incidence (per 1000 person-years) of type 2 diabetes from 2006 to 2015 across the 30 most common occupations in women in Sweden

The age-standardised incidence of diabetes per 1000 person-years was 5.19 (95% CI 5.17, 5.22) overall, and higher in men 6.36 (6.33, 6.40) than in women 4.03 (4.00, 4.06). Comparison of the 30 most common occupations among men indicated that incidence was highest among manufacturing workers (9.41), motor vehicle drivers (9.32), agricultural and other mobile plant operators (8.31), personal carers (8.17) and stores and transport clerks (7.87), and lowest among university teachers (3.44), architects and civil engineers (3.83) (Fig. 2 and ESM Table 1). Corresponding estimates among women show that the incidence was highest among manufacturing labourers (7.20), cleaners (6.18), kitchen assistants (5.65), cooks, waitresses and housekeepers (5.01), and personal carers, which includes child-care workers, attendants in psychiatric care and assistant nurses (5.00), and lowest among physiotherapists and dental hygienists (2.20) and writers, creative or performing artists (2.27) (Fig. 3 and ESM Table 2). SIR calculations (ESM Tables 1 and 2) showed that, compared with the total working population, manufacturing workers had SIRs of 1.49 (95% CI 1.44, 1.55) (men) and 1.80 (95% CI 1.72, 1.89) (women). In contrast, a 46% reduced incidence was seen in male college and university teachers and a 45% reduced incidence was found in female physiotherapists and dental hygienists. Estimation of population attributable risk percentage indicated that 46% of all cases of type 2 diabetes in men and 45% in women would be eliminated if the total working population had the same incidence as college and university teachers (men) or physiotherapists and dental hygienists (women) (ESM Tables 1 and 2).

The occupations with highest incidence were those characterised by low SES according to the classification used by Statistics Sweden [18], and included skilled and unskilled manual workers (occupations that require less than 12 years of education). However, incidence varied dramatically across occupations in the low-SES strata (ESM Tables 1 and 2); for example, in men, manufacturing labourers had an incidence of 9.41 compared with 5.84 among construction workers. Similarly, female manufacturing workers had an incidence of 7.20 compared with 3.95 in retail sales workers. Individuals without an occupation recorded between 2001 and 2013 had an age-standardised incidence of 8.29 overall, 8.89 in men and 7.72 in women.

A complete overview of incidence and prevalence of type 2 diabetes across all occupations classified by SSYK96 is given in ESM Table 5. These analyses confirm that manufacturing workers, motor vehicle drivers and cleaners are among the top ten occupations in terms of type 2 diabetes incidence and prevalence, and show a clear socioeconomic gradient in the association between occupation and occurrence of type 2 diabetes.

### BMI, smoking and physical fitness by occupation

**Fig. 4:**
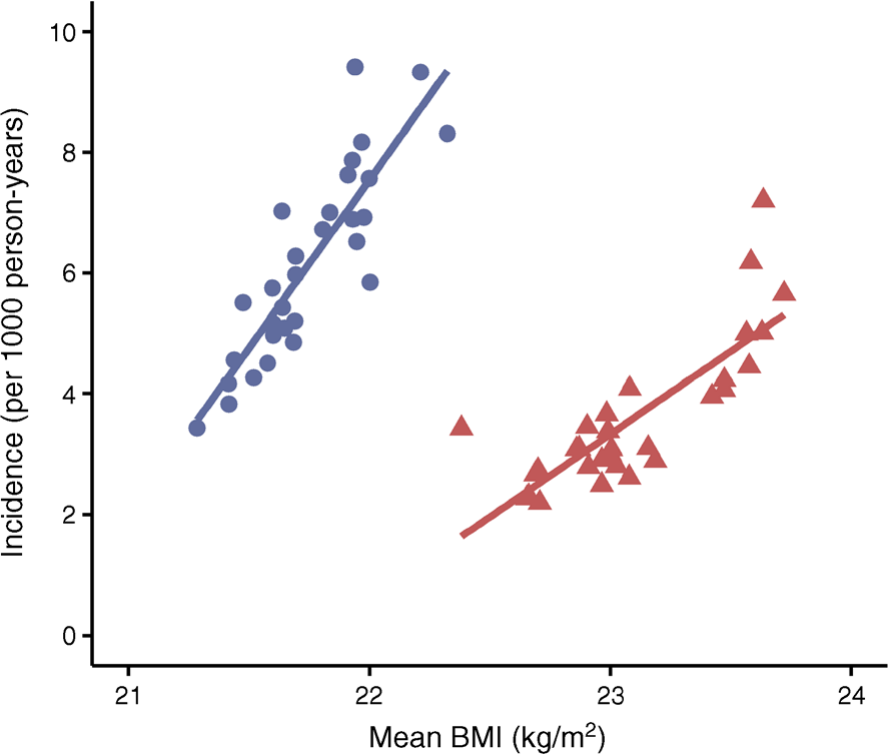
Mean BMI and age-standardised incidence (per 1000 person-years) of type 2 diabetes from 2006 to 2015 across the 30 most common occupations in Swedish men and women. Blue circles, men; red triangles, women

As shown in Fig. 4, there was a strong, positive association between incidence of type 2 diabetes and mean BMI within the 30 most frequent occupations in men and women. In men, data from the Military Conscription Register show that the prevalence of being overweight at the mean age of 18 is high in men who will subsequently be working as motor vehicle drivers (16.3%), agricultural and other mobile plant operators (16.8%) and manufacturing workers (14.1%), whereas in future college and university teachers, only 6.5% are overweight at conscription (ESM Table 6). A similar, inverse gradient is seen for physical fitness measured by *W*_max_. In women, data from the Medical Birth Register show that 29–30% of cleaners, manufacturing workers and kitchen assistants were overweight in the first trimester (registered at mean age 29 years), and 24–30% were smokers (ESM Table 7). Corresponding estimates in writers, creative or performing artists, physiotherapists and dental hygienists were 18% for being overweight and 6% for smoking.

## Discussion

### Principal findings

We found striking differences between common occupational groups in Sweden, both in terms of prevalence of type 2 diabetes, which ranged from 2.5% to 8.8% in men and from 1.2% to 6.4% in women, and incidence, which ranged from 3.44 to 9.41 per 1000 person-years in men and from 2.20 to 7.20 per 1000 person-years in women. The highest prevalence and incidence were seen among manufacturing workers, motor vehicle drivers and cleaners, whereas civil engineers, architects, teachers, physiotherapists, writers, managers and computer scientists had the lowest incidence as well as prevalence.

The association between occupation and type 2 diabetes coincided with vast differences in the prevalence of lifestyle risk factors. With the register data, we could show that individuals in high-risk occupations were more likely to be overweight, smoke and have lower physical fitness than those in low-risk occupations, and this most likely contributes to a high prevalence and incidence of type 2 diabetes. Interestingly, these differences were already seen at young ages, at the time of first pregnancy in women, and in men at the time of military conscription, which was at the age of entering the labour market. These findings indicate that job title is a risk indicator rather than a causal factor in relation to type 2 diabetes. In line with this, previous studies have shown that the excess risk of type 2 diabetes in individuals with low SES is mainly attributable to adverse lifestyle factors, primarily obesity [19, 20]. Of note, the lifestyle information was based on historical data, and the differences in prevalence of lifestyle factors may be even larger at older ages. It should also be noted that this information was only available for about half of the study population. Still, this finding fits with previous observations showing that individuals who develop type 2 diabetes differ metabolically from those who do not, up to 25 years prior to diagnosis [21]. On top of the strong lifestyle selection into different occupations, it is possible that working life adds more risk factors in the form of long sitting times, irregular working hours and stress. This is certainly true for motor vehicle drivers, which includes bus, taxi and lorry drivers. However, engineers and computing professionals are also likely to be sedentary, yet they are at very low risk of type 2 diabetes. Manufacturing work on the other hand may be associated with less sitting than white collar work, but may be stressful and include shift work. The role of working conditions in the promotion of type 2 diabetes is an important area to explore further. In addition, individuals with low SES may be exposed to poorer living conditions, relating to social support, housing and material conditions, which may make them more vulnerable to developing type 2 diabetes [22]. In this context, it is also important to note that we observed that individuals without an occupation, e.g. those who are unemployed or on disability pension, had very high incidence and prevalence of type 2 diabetes.

### Strengths and weaknesses of the study

The main strength of this study is the use of register data which allow us to conduct a nationwide study in Sweden, making this by far the most comprehensive overview of the relationship between occupation and type 2 diabetes. There is no non-response since the registries include all Swedish citizens. Cases of diabetes were identified through the Prescription Register, which can be expected to be complete for pharmacologically treated diabetes, and through the Patient Register. This means that we will miss cases treated non-pharmacologically, if they have not received specialist care, since primary care is not covered by the Swedish patient registries. This will not bias our results as long as treatment strategy and occupation are uncorrelated; however, it does mean that we underestimate diabetes occurrence; in Sweden 20–25% of all patients with type 2 diabetes are treated exclusively by lifestyle modification [23]. Undiagnosed cases will also be missed and it is possible that the proportion of such cases differs across occupations. In this context, it is noteworthy that Sweden has a universal healthcare system which is available to all citizens at minimal cost. We used ICD codes or age ≥ 35 at the time of the first prescription of diabetes medication as indicators of type 2 diabetes. This implies that we may have included some individuals with autoimmune diabetes, but this misclassification is likely to be minor since type 2 diabetes is by far the most common form of diabetes in this age group. Some people changed their occupation during follow-up; to reduce such misclassification, a person had to be registered in a particular occupation for at least two consecutive years to be classified into that occupational group. It is noteworthy that individuals in low-SES occupations may change occupation more often than those in high SES occupations, since the latter are more specialised; whether this contributes to excess risk of type 2 diabetes is not clear. Regarding generalisability, it should be noted that diabetes prevalence in Sweden is on a par with observations from other European countries [1]. Hence, it seems reasonable to assume that there are occupational groups with similarly high prevalence of type 2 diabetes in other Westernised countries. Still, to what extent our results are generalisable to countries with different healthcare systems and larger socioeconomic inequality is not clear.

### Strengths and weaknesses in relation to other studies

It is well known from previous studies that low SES is associated with excess risk of type 2 diabetes [6]. In support thereof, we find that the occupations with the highest incidence also fall into the low-SES group. However, we also show that job title is a much more specific indicator of type 2 diabetes risk than SES alone, since we find that, even across occupations characterised by low SES, incidence varies substantially. Previous data on diabetes prevalence in relation to occupation are limited. In fact, the most complete previous report is a US Gallup survey based on 90,000 US workers and conducted in 2016 [10]. Consistent with our findings, it showed that prevalence was highest among transportation workers (10.3%) and lowest among physicians (5.1%). In addition, and in line with our findings, this survey revealed that transportation workers had a high prevalence of obesity and smoking. This report [10] was based on self-reports and may not be representative of the total working populations due to non-response. In an Australian cross-sectional study based on >500,000 employees, the prevalence of a composite measure of type 2 diabetes risk was investigated across 20 different industries [11]. Consistent with our findings, the proportion of employees with high risk of diabetes was highest in the manufacturing industry and in transport, postal and warehousing industries. With regard to incidence, there is a lack of studies, and as far as we know the present study is to date the most complete study on incidence of type 2 diabetes in relation to occupation.

### Meaning of the study and unanswered questions

Many Western countries are looking to increase retirement age in order to secure the fiscal stability of the pension system in response to a growing proportion of older people in the population. The high prevalence of diabetes is important to consider in this context. We find that there are occupations, such as manufacturing workers and motor vehicle drivers, where more than 7% of employees have type 2 diabetes, and prevalence increases with age. As an example, more than one in seven of all male manufacturing workers aged ≥55 years have type 2 diabetes. To what extent this is reflected in high morbidity and sickness absence and disability pension is not known. Recent Swedish studies indicate that low SES is associated with increased risk of diabetes complications [24] and disability related to diabetes [25], but few studies have explored the risk of comorbidity and work disability across different occupational groups [26]. It seems possible that workplace factors such as irregular working hours, shift work, stress and physically strenuous work may hinder optimal self-management and contribute to excess risk of complications. This is an important area for future studies since, from a societal as well as individual perspective, it is important to adapt working life so that people with diabetes can manage their disease adequately, thereby reducing the risk of complications. Corresponding data from other countries are currently lacking, but are important to produce and evaluate in relation to proposals regarding retirement age since diabetes may potentially hinder prolonged working life.

To reduce the future diabetes burden, it is crucial to curb the inflow of new patients. The preventive potential of type 2 diabetes is substantial; a recent study based on >400,000 individuals estimated that the combination of healthy BMI, waist/hip ratio and diet together with non-smoking could prevent three-quarters of all cases of type 2 diabetes [27]. Furthermore, intervention studies have consistently shown that it is possible to reduce incidence in high-risk groups by lifestyle modification; data from the Finnish Diabetes Prevention Programme [28] and the US Diabetes Prevention Program [29] show a 40% risk reduction following intervention focusing on a healthy diet, weight reduction and physical activity. In the present study, we aimed to explore whether job title can be used as a risk indicator of type 2 diabetes, allowing us to identify groups that may be suitable for targeted interventions. Such information could hopefully inspire employers to implement diabetes prevention programmes tailored to their workforces. Evaluations of workplace interventions have shown promising results; a recent review, based primarily on studies conducting in the USA, demonstrates positive effects primarily on weight from translation of the US Diabetes Prevention Program to workplace settings [30]. Our study shows that there are occupational groups, including motor vehicle drivers, manufacturing workers and cleaners, where the incidence of type 2 diabetes is increased threefold and, furthermore, that employees in these occupations already have a higher prevalence of lifestyle risk factors in their twenties and thirties. If interventions could reduce weight and smoking and increase physical activity among employees in these occupations, major health and productivity gains may be made.

## Electronic supplementary material

ESM (PDF 607 kb)

## Data Availability

The data that support the findings of this study are available from Statistics Sweden and the Swedish National Board of Health and Welfare; however, restrictions apply to the availability of these data, which were used under license for the current study, and so they are not publicly available.

## Abbreviations

LISA: Longitudinal Integrated Database for Health Insurance and Labour Market Studies
SES: Socioeconomic status
SIR: Standardised incidence rate ratio
SSYK96: Swedish Standard Classification of Occupations 1996
*W*_max_: Maximal aerobic workload
