# Supplementary material for: Incidence and prevalence of type 2 diabetes by occupation: results from all Swedish employees
Source: Diabetologia. 2019 Sep 17;63(1):95–103. doi: 10.1007/s00125-019-04997-5 (PMC6890587; doi:10.1007/s00125-019-04997-5)

ESM Table 1. Incidence (per 1000 person-years) 2006-2015 and prevalence (per 100) in 2013 of type 2 diabetes among the 30 most common occupations in Swedish men.

Occupation	No. Cases	Person-years	Age-standardized Incidence (95% CI)	SIR (95% CI)	No. Cases	Prevalence (95% CI)	Age-standardized Prevalence (95% CI)	PAR %
**Manufacturing labourers[1]**	3 263	359 768	9.41 (9.09-9.74)	1.49 (1.44-1.55)	1 160	7.75 (7.32-8.17)	7.77 (7.35-8.19)	1.75%
**Motor-vehicle drivers[1]**	10 762	1 134 058	9.32 (9.15-9.50)	1.47 (1.44-1.50)	8 143	8.77 (8.59-8.96)	8.00 (7.84-8.17)	5.42%
**Agricultural and other mobile plant operators[1]**	3 376	408 776	8.31 (8.03-8.59)	1.31 (1.26-1.35)	2 170	7.21 (6.92-7.50)	6.97 (6.69-7.24)	1.62%
**Personal carers[1b]**	4 852	696 143	8.17 (7.93-8.41)	1.34 (1.30-1.38)	4 066	7.12 (6.91-7.33)	7.46 (7.24-7.68)	2.68%
**Stores and transport clerks**	4 993	714 456	7.87 (7.65-8.09)	1.25 (1.21-1.28)	2 260	6.12 (5.87-6.36)	6.43 (6.17-6.68)	2.58%
**Machine operators[1]**	1 854	295 306	7.63 (7.25-8.00)	1.22 (1.17-1.28)	1 001	5.75 (5.40-6.09)	6.23 (5.86-6.60)	1.00%
**Metal- and mineral-products machine operators[1]**	4 020	587 463	7.57 (7.33-7.81)	1.20 (1.16-1.23)	1 845	6.09 (5.82-6.36)	6.16 (5.89-6.43)	1.97%
**Office clerks**	2 314	340 379	7.03 (6.74-7.32)	1.10 (1.06-1.15)	1 276	7.14 (6.76-7.51)	6.70 (6.35-7.05)	0.99%
**Retail salespersons[1]**	3 826	709 113	7.01 (6.78-7.24)	1.10 (1.06-1.13)	2 431	5.35 (5.14-5.56)	6.03 (5.80-6.26)	2.06%
**Metal moulders. welders and sheet-metal workers[1]**	2 993	463 715	6.93 (6.68-7.17)	1.09 (1.05-1.13)	1 386	5.29 (5.02-5.56)	5.37 (5.10-5.64)	1.32%
**Assemblers[1]**	3 074	551 473	6.89 (6.64-7.15)	1.10 (1.06-1.13)	1 505	5.33 (5.07-5.59)	5.85 (5.56-6.13)	1.55%
**Building finishers and related trades workers[1]**	7 692	1 117 810	6.72 (6.57-6.87)	1.06 (1.03-1.08)	4 791	5.72 (5.56-5.88)	5.27 (5.13-5.41)	2.99%
**Machinery mechanics and fitters[1]**	4 315	684 991	6.51 (6.32-6.71)	1.02 (0.99-1.05)	2 371	5.36 (5.15-5.57)	5.26 (5.06-5.46)	1.71%
**Total working population**	123 114	19 345 377	6.36 (6.33-6.40)	1.00 (0.99-1.01)	93 997	5.19 (5.15-5.22)	5.19 (5.15-5.22)	46.04%
**Electrical and electronic equipment mechanics[1]**	2 715	455 802	6.28 (6.04-6.52)	0.98 (0.95-1.02)	1 257	5.48 (5.18-5.77)	5.02 (4.75-5.29)	1.05%
**Managers of small enterprises**	5 986	930 234	5.97 (5.81-6.12)	0.94 (0.92-0.96)	2 949	5.29 (5.11-5.48)	4.85 (4.68-5.01)	1.91%
**Construction workers[1]**	6 305	1 102 673	5.84 (5.70-5.99)	0.92 (0.89-0.94)	3 663	4.13 (4.00-4.26)	4.14 (4.01-4.27)	2.16%
**Computer technicians and data operators**	1 738	436 861	5.75 (5.43-6.06)	0.90 (0.86-0.94)	945	3.64 (3.41-3.86)	4.82 (4.51-5.13)	0.82%
**Accountants**	1 795	314 650	5.51 (5.25-5.76)	0.87 (0.83-0.91)	858	5.53 (5.17-5.89)	4.96 (4.63-5.28)	0.53%
**Finance and sales associate professionals**	7 921	1 594 203	5.43 (5.31-5.55)	0.84 (0.82-0.86)	4 311	4.01 (3.89-4.12)	4.30 (4.18-4.43)	2.59%

**Elementary school teachers**	1 451	284 469	5.20 (4.93-5.47)	0.81 (0.77-0.86)	697	4.43 (4.11-4.75)	4.33 (4.02-4.65)	0.41%
**Engineers and technicians**	8 908	1 719 893	5.16 (5.05-5.26)	0.81 (0.79-0.83)	4 077	4.26 (4.13-4.38)	4.25 (4.12-4.37)	2.40%
**Public service administrative professionals**	2 151	355 391	5.08 (4.86-5.30)	0.81 (0.78-0.85)	972	4.80 (4.50-5.09)	3.90 (3.66-4.15)	0.47%
**Directors and chief executives**	1 988	347 576	4.97 (4.75-5.19)	0.78 (0.75-0.82)	934	4.06 (3.80-4.31)	3.88 (3.64-4.12)	0.43%
**Production and operations managers**	3 864	739 382	4.85 (4.69-5.00)	0.77 (0.74-0.79)	1 604	3.53 (3.36-3.70)	3.59 (3.42-3.77)	0.85%
**Computer scientists**	2 741	881 965	4.56 (4.36-4.75)	0.68 (0.65-0.70)	1 600	2.46 (2.34-2.58)	3.61 (3.43-3.78)	0.80%
**Senior high school teachers**	1 867	372 260	4.50 (4.30-4.71)	0.71 (0.68-0.74)	1 065	4.31 (4.05-4.56)	3.77 (3.55-4.00)	0.32%
**Specialist managers**	3 671	876 827	4.27 (4.12-4.41)	0.66 (0.64-0.68)	1 371	2.81 (2.66-2.96)	3.16 (3.00-3.33)	0.59%
**Business professionals**	3 199	781 043	4.16 (4.02-4.31)	0.65 (0.63-0.67)	1 555	3.42 (3.25-3.59)	3.37 (3.21-3.54)	0.47%
**Architects and civil engineers**	2 786	837 665	3.83 (3.68-3.97)	0.59 (0.56-0.61)	1 500	2.80 (2.66-2.94)	3.25 (3.09-3.41)	0.27%
**College and university teachers**	794	262 374	3.44 (3.20-3.68)	0.54 (0.50-0.57)	401	2.65 (2.40-2.91)	2.69 (2.43-2.95)	Reference

[1] *Occupations classified as being in the lowest socio-economic status group. i.e. "skilled and unskilled manual workers" according to Statistics Sweden.* [b]*Personal carers includes child-care workers, assistant nurses, hospital ward assistants, home-based personal care, attendants in psychiatric care and dental nurses.*

ESM Table 2. Incidence (per 1000 person-years) 2006-2015 and prevalence (per 100) in 2013 of type 2 diabetes among the 30 most common occupations in Swedish women.

Occupation	No. Cases	Person-years	Age-standardized Incidence (95% CI)	SIR (95% CI)	No. Cases	Prevalence (95% CI)	Age-standardized prevalence (95% CI)	PAR %
**Manufacturing labourers[1]**	1 743	244 236	7.20 (6.86-7.54)	1.80 (1.72-1.89)	634	6.42 (5.94-6.91)	5.93 (5.49-6.38)	1.55%
**Cleaners[1]**	5 562	885 336	6.18 (6.02-6.34)	1.53 (1.49-1.57)	3 286	5.09 (4.92-5.26)	4.90 (4.73-5.06)	4.48%
**Kitchen assistants[1]**	3 687	659 950	5.65 (5.47-5.84)	1.39 (1.35-1.44)	1 869	5.45 (5.21-5.69)	5.10 (4.88-5.33)	2.90%
**Cooks, waitresses and housekeepers[1]**	2 247	471 604	5.01 (4.80-5.22)	1.24 (1.19-1.29)	1 102	4.06 (3.82-4.29)	4.13 (3.90-4.37)	1.68%
**Personal carers[1b]**	23 898	4 954 782	5.00 (4.93-5.06)	1.25 (1.23-1.26)	16 743	4.31 (4.25-4.38)	4.18 (4.12-4.24)	17.65%
**Cashiers, tellers and related clerks[1]**	1 162	266 063	4.45 (4.19-4.71)	1.09 (1.03-1.15)	381	4.17 (3.76-4.58)	4.12 (3.71-4.54)	0.77%
**Social work associate professionals[1]**	1 175	319 264	4.23 (3.98-4.48)	1.05 (0.99-1.11)	585	3.12 (2.87-3.37)	3.43 (3.16-3.71)	0.83%
**Office clerks**	5 910	1 357 026	4.08 (3.98-4.19)	1.02 (0.99-1.04)	2 540	3.83 (3.68-3.98)	3.38 (3.25-3.51)	3.26%
**Client information clerks**	1 873	527 156	4.06 (3.88-4.25)	1.00 (0.96-1.05)	857	3.24 (3.03-3.46)	3.32 (3.11-3.54)	1.25%
**Total working population**	78 603	19 493 239	4.03 (4.00-4.06)	1.00 (0.99-1.01)	56 134	3.17 (3.14-3.20)	3.17 (3.14-3.20)	45.38%
**Retail salespersons[1]**	4 390	1 263 785	3.95 (3.83-4.07)	0.97 (0.94-1.00)	2 179	2.86 (2.74-2.98)	3.02 (2.89-3.14)	2.81%
**Numerical clerks**	3 466	904 243	3.66 (3.54-3.79)	0.91 (0.88-0.95)	1 571	3.35 (3.19-3.51)	2.91 (2.76-3.05)	1.68%
**Office secretaries and data entry operators**	2 571	685 184	3.46 (3.32-3.59)	0.85 (0.82-0.89)	1 119	3.55 (3.34-3.75)	3.05 (2.87-3.23)	1.10%
**Health professionals (except nursing)[c]**	821	251 156	3.43 (3.19-3.67)	0.90 (0.83-0.96)	588	2.71 (2.50-2.93)	2.89 (2.66-3.12)	0.39%
**Managers of small enterprises**	1 396	421 910	3.38 (3.20-3.56)	0.83 (0.79-0.87)	496	2.18 (1.99-2.37)	2.29 (2.09-2.49)	0.64%
**Engineers and technicians**	844	344 944	3.10 (2.88-3.32)	0.74 (0.69-0.79)	345	1.90 (1.70-2.10)	2.26 (2.02-2.50)	0.39%
**Accountants**	3 198	1 041 511	3.09 (2.98-3.20)	0.77 (0.74-0.79)	1 578	2.63 (2.51-2.76)	2.51 (2.39-2.63)	1.18%
**Nurses**	2 150	745 347	3.08 (2.95-3.21)	0.76 (0.73-0.79)	1 318	2.44 (2.31-2.57)	2.41 (2.28-2.54)	0.84%
**Public service administrative professionals**	1 492	486 201	3.08 (2.92-3.24)	0.76 (0.72-0.80)	685	2.21 (2.04-2.37)	2.20 (2.04-2.36)	0.54%
**Psychologists and social workers**	1 000	339 377	2.98 (2.79-3.17)	0.74 (0.70-0.79)	617	2.42 (2.24-2.61)	2.43 (2.24-2.61)	0.34%
**Finance and sales associate professionals**	2 385	1 003 739	2.91 (2.79-3.03)	0.70 (0.67-0.73)	1 080	1.73 (1.63-1.83)	2.01 (1.89-2.13)	0.91%
**Preschool and recreation teachers**	2 128	860 863	2.89 (2.76-3.02)	0.70 (0.67-0.73)	1 494	2.12 (2.01-2.22)	2.23 (2.12-2.34)	0.76%
**Computer scientists**	515	289 962	2.81 (2.48-3.13)	0.63 (0.58-0.69)	300	1.47 (1.31-1.64)	2.02 (1.77-2.27)	0.23%
**Production and operations managers**	1 155	425 016	2.79 (2.62-2.96)	0.69 (0.65-0.73)	766	1.99 (1.85-2.13)	2.10 (1.95-2.25)	0.32%
**Midwifes and specialist nurses**	1 351	472 416	2.74 (2.59-2.89)	0.68 (0.64-0.71)	622	2.19 (2.02-2.36)	2.10 (1.94-2.27)	0.33%

**Business professionals**	1 865	846 841	2.67 (2.54-2.79)	0.63 (0.61-0.66)	887	1.67 (1.56-1.78)	1.97 (1.84-2.10)	0.51%
**Elementary school teachers**	2 162	859 285	2.62 (2.51-2.73)	0.65 (0.62-0.68)	1 144	1.98 (1.86-2.09)	2.08 (1.96-2.20)	0.46%
**Senior high school teachers**	1 134	437 258	2.49 (2.35-2.64)	0.62 (0.58-0.65)	565	1.98 (1.82-2.14)	1.92 (1.76-2.08)	0.16%
**Specialist managers**	632	356 058	2.30 (2.10-2.50)	0.52 (0.48-0.56)	265	1.17 (1.03-1.31)	1.62 (1.41-1.82)	0.05%
**Writers, creative or performing artists**	488	288 759	2.27 (2.06-2.48)	0.53 (0.48-0.58)	264	1.29 (1.14-1.44)	1.67 (1.46-1.88)	0.03%
**Physiotherapists and dental hygienists**	728	351 091	2.20 (2.04-2.36)	0.55 (0.51-0.59)	434	1.60 (1.45-1.75)	1.63 (1.48-1.78)	Reference

[1] Occupations classified as being in the lowest socio-economic status group. i.e. "skilled and unskilled manual workers" according to Statistics Sweden. [b]Personal carers includes child-care workers, assistant nurses, hospital ward assistants, home-based personal care, attendants in psychiatric care and dental nurses .[c] Health professionals includes physicians, veterinarians, dentists, pharmacists and speech therapists.

ESM Table 3. Prevalence (per 100) of type 2 diabetes in men age ≥55 years among the 30 most common occupations in Swedish men in 2013.

Occupation	No. Cases	No. population	Prevalence	95% CI
**Manufacturing labourers[1]**	780	5 242	14.88	13.92-15.84
**Motor-vehicle drivers[1]**	5 330	37 459	14.23	13.88-14.58
**Office clerks**	927	7 078	13.10	12.31-13.88
**Agricultural and other mobile plant operators[1]**	1 453	11 098	13.09	12.46-13.72
**Personal carers[1b]**	2 350	18 391	12.78	12.30-13.26
**Stores and transport clerks**	1 473	12 035	12.24	11.65-12.82
**Metal- and mineral-products machine operators[1]**	1 168	10 114	11.55	10.93-12.17
**Retail salespersons[1]**	1 504	13 408	11.22	10.68-11.75
**Machine operators[1]**	580	5 251	11.05	10.20-11.89
**Assemblers[1]**	909	8 398	10.82	10.16-11.49
**Metal moulders. welders and sheet-metal workers[1]**	931	8 783	10.60	9.96-11.24
**Machinery mechanics and fitters[1]**	1 619	15 849	10.22	9.74-10.69
**Electrical and electronic equipment mechanics[1]**	927	9 117	10.17	9.55-10.79
**Building finishers and related trades workers[1]**	3 371	33 217	10.15	9.82-10.47
**Managers of small enterprises**	651	6 420	10.14	9.40-10.88
**Accountants**	2 182	21 665	10.07	9.67-10.47
**Total working population**	63 997	635 246	10.07	10.00-10.15
**Computer technicians and data operators**	542	5 803	9.34	8.59-10.09
**Finance and sales associate professionals**	3 113	34 095	9.13	8.82-9.44
**Engineers and technicians**	2 957	33 322	8.87	8.57-9.18
**Public service administrative professionals**	793	9 239	8.58	8.01-9.15
**Elementary school teachers**	511	6 072	8.42	7.72-9.11
**Directors and chief executives**	697	8 397	8.30	7.71-8.89
**Construction workers[1]**	2 488	30 347	8.20	7.89-8.51
**Production and operations managers**	1 159	15 544	7.46	7.04-7.87
**Computer scientists**	990	13 301	7.44	7.00-7.89
**Senior high school teachers**	795	10 850	7.33	6.84-7.82
**Business professionals**	1 181	16 346	7.23	6.83-7.62
**Architects and civil engineers**	1 057	15 346	6.89	6.49-7.29
**Specialist managers**	967	14 280	6.77	6.36-7.18
**College and university teachers**	287	5 309	5.41	4.80-6.01

[1] Occupations classified as being in the lowest socio-economic status group. i.e. "skilled and unskilled manual workers" according to Statistics Sweden. [b]Personal carers includes child-care workers, assistant nurses, hospital ward assistants, home-based personal care, attendants in psychiatric care and dental nurses.

ESM Table 4. Prevalence (per 100) of type 2 diabetes in women age ≥55 years among the 30 most common occupations in Swedish women in 2013.

Occupation	No. Cases	No. population	Prevalence	95% CI
Manufacturing labourers[1]	430	4 037	10.65	9.70-11.60
Kitchen assistants[1]	1 197	13 832	8.65	8.19-9.12
Cleaners[1]	2 041	24 577	8.30	7.96-8.65
Cashiers, tellers and related clerks[1]	270	3 346	8.07	7.15-8.99
Cooks, waitresses and housekeepers[1]	742	9 345	7.94	7.39-8.49
Personal carers[1b]	10 246	148 687	6.89	6.76-7.02
Office clerks	1 868	28 620	6.53	6.24-6.81
Social work associate professionals[1]	360	5 698	6.32	5.69-6.95
Client information clerks	536	9 111	5.88	5.40-6.37
Total working population	36 076	634 616	5.68	5.63-5.74
Retail salespersons[1]	1 382	24 515	5.64	5.35-5.93
Numerical clerks	1 135	20 737	5.47	5.16-5.78
Office secretaries and data entry operators	789	14 625	5.39	5.03-5.76
Accountants	1 088	23 224	4.68	4.41-4.96
Engineers and technicians	226	4 836	4.67	4.08-5.27
Managers of small enterprises	329	7 372	4.46	3.99-4.93
Psychologists and social workers	410	9 409	4.36	3.95-4.77
Nurses	858	20 533	4.18	3.90-4.45
Health professionals (except nursing)[c]	288	7 155	4.03	3.57-4.48
Public service administrative professionals	456	11 362	4.01	3.65-4.37
Finance and sales associate professionals	689	17 208	4.00	3.71-4.30
Business professionals	575	14 532	3.96	3.64-4.27
Elementary school teachers	762	19 796	3.85	3.58-4.12
Computer scientists	146	3 874	3.77	3.17-4.37
Preschool and recreation teachers	898	24 174	3.71	3.48-3.95
Production and operations managers	481	13 146	3.66	3.34-3.98
Senior high school teachers	379	10 757	3.52	3.17-3.87
Midwifes and specialist nurses	401	11 659	3.44	3.11-3.77
Writers, creative or performing artists	153	4 652	3.29	2.78-3.80
Specialist managers	149	4 745	3.14	2.64-3.64
Physiotherapists and dental hygienists	262	9 528	2.75	2.42-3.08

[1] Occupations classified as being in the lowest socio-economic status group. i.e. "skilled and unskilled manual workers" according to Statistics Sweden. [b]Personal carers includes child-care workers, assistant nurses, hospital ward assistants, home-based personal care, attendants in psychiatric care and dental nurses.

ESM Table 5. Age and sex-standardized incidence (per 1000 person-years) 2006-2015 and prevalence (per 100) 2013 of type 2 diabetes in men and women combined across all occupations in Sweden.

Occupation	No. incident cases	Person-years	Incidence (95% CI)	SIR (95% CI)	No. prevalent cases	Prevalence (95% CI)
Sales and services elementary occupations	3 688	377 108	9.33 (9.01-9.65)	1.78 (1.72-1.84)	3 559	10.93 (10.59-11.27)
Mining and construction labourers	255	40 503	8.55 (4.37-12.72)	1.22 (1.08-1.38)	154	4.75 (4.02-5.48)
Street vendors and market salespersons	47	4 951	8.33 (5.79-10.87)	1.63 (1.20-2.16)	75	9.45 (7.41-11.48)
Manufacturing labourers	5 006	604 004	8.30 (8.07-8.54)	1.59 (1.55-1.63)	1 794	7.22 (6.90-7.54)
Mineral-processing-plant operators	149	21 309	8.28 (5.27-11.29)	1.24 (1.05-1.45)	87	6.33 (5.04-7.62)
Industrial-robot operators	82	13 791	8.10 (5.63-10.56)	1.33 (1.06-1.66)	37	4.60 (3.15-6.04)
Cleaners	7 477	1 113 879	8.00 (7.75-8.24)	1.56 (1.53-1.60)	4 613	5.63 (5.47-5.79)
Kitchen assistants	4 905	836 537	7.71 (7.38-8.04)	1.46 (1.42-1.50)	2 573	5.73 (5.51-5.94)
Motor-vehicle drivers	11 281	1 229 449	7.59 (7.32-7.86)	1.47 (1.44-1.50)	8 504	8.49 (8.31-8.66)
Food and related products machine operators	1 871	309 780	7.06 (6.72-7.40)	1.37 (1.31-1.44)	813	5.52 (5.15-5.89)
Food processing and related trades workers	948	145 129	7.00 (6.46-7.53)	1.45 (1.36-1.54)	548	6.12 (5.62-6.62)
Housekeeping and restaurant services workers	3 724	713 956	6.99 (6.71-7.26)	1.30 (1.26-1.34)	2 185	4.80 (4.60-4.99)
Metal-processing-plant operators	1 615	198 797	6.99 (6.50-7.48)	1.36 (1.29-1.43)	908	7.10 (6.66-7.55)
Garbage collectors and related labourers	1 115	135 370	6.98 (6.25-7.72)	1.36 (1.28-1.44)	626	7.29 (6.74-7.84)
Wood-processing- and papermaking-plant operators	1 934	250 583	6.94 (6.48-7.41)	1.30 (1.24-1.36)	1 077	6.91 (6.51-7.31)
Chemical-processing-plant operators	548	92 692	6.87 (6.05-7.69)	1.20 (1.11-1.31)	279	5.34 (4.73-5.95)
Rubber- and plastic-products machine operators	1 182	193 926	6.65 (6.24-7.06)	1.28 (1.21-1.35)	583	5.69 (5.24-6.14)
Glass, ceramics and related plant operators	144	21 900	6.59 (5.32-7.86)	1.28 (1.08-1.51)	62	5.15 (3.90-6.39)
Personal carers	28 750	5 650 925	6.58 (6.46-6.70)	1.26 (1.25-1.27)	20 809	4.67 (4.61-4.74)
Metal- and mineral-products machine operators	4 473	675 666	6.55 (6.27-6.84)	1.21 (1.18-1.25)	2 019	5.87 (5.62-6.12)
Transport labourers and freight handlers	1 085	167 706	6.53 (5.89-7.16)	1.22 (1.15-1.29)	658	6.15 (5.70-6.61)
Doorkeepers, newspaper and package deliverers and related workers	2 254	297 234	6.51 (6.21-6.82)	1.23 (1.18-1.28)	1 192	7.20 (6.80-7.59)
Other machine operators and assemblers	2 466	426 856	6.42 (6.13-6.70)	1.24 (1.19-1.29)	1 282	5.29 (5.01-5.57)
Textile-, fur- and leather-products machine operators	796	125 487	6.41 (5.93-6.89)	1.24 (1.15-1.33)	389	6.12 (5.53-6.71)
Chemical-products machine operators	994	182 175	6.30 (5.87-6.73)	1.21 (1.13-1.29)	512	4.91 (4.49-5.32)
Cashiers, tellers and related clerks	1 490	322 410	6.23 (5.76-6.70)	1.12 (1.06-1.18)	511	4.49 (4.11-4.88)
Miners, shot firers, stonecutters and carvers	281	39 646	6.22 (4.22-8.22)	1.15 (1.02-1.29)	175	5.49 (4.70-6.28)

**Printing-, binding- and paper-products machine operators**	1 186	180 223	6.21 (5.79-6.62)	1.18 (1.11-1.25)	625	5.97 (5.52-6.42)
**Stores and transport clerks**	5 857	946 214	6.19 (6.00-6.38)	1.22 (1.19-1.25)	2 631	5.49 (5.29-5.70)
**Construction workers**	6 408	1 123 041	6.14 (5.44-6.84)	0.92 (0.90-0.95)	3 787	4.15 (4.02-4.28)
**Blacksmiths, tool-makers and related trades workers**	1 932	284 014	6.10 (5.65-6.55)	1.11 (1.06-1.16)	571	5.87 (5.40-6.34)
**Agricultural and other mobile-plant operators**	3 435	425 815	6.06 (5.54-6.58)	1.30 (1.26-1.34)	2 209	7.03 (6.74-7.31)
**Client information clerks**	2 515	631 274	5.99 (5.65-6.32)	1.06 (1.02-1.11)	1 281	3.93 (3.72-4.15)
**Wood-products machine operators**	1 571	263 751	5.96 (5.57-6.36)	1.07 (1.02-1.12)	499	5.29 (4.84-5.75)
**Fishery workers, hunters and trappers**	140	16 723	5.95 (2.84-9.07)	1.13 (0.95-1.34)	85	5.87 (4.66-7.08)
**Social work associate professionals**	2 252	489 615	5.95 (5.68-6.23)	1.13 (1.08-1.18)	1 175	4.18 (3.94-4.41)
**Assemblers**	3 977	762 677	5.90 (5.69-6.11)	1.12 (1.09-1.16)	1 951	5.01 (4.79-5.23)
**Religious professionals**	296	42 278	5.82 (5.09-6.56)	1.12 (0.99-1.25)	210	5.83 (5.07-6.60)
**Travel attendants and related workers**	366	86 886	5.76 (5.13-6.39)	1.05 (0.95-1.17)	214	3.85 (3.35-4.36)
**Protective services workers**	1 712	348 425	5.73 (5.40-6.07)	1.08 (1.03-1.13)	977	4.40 (4.13-4.67)
**Building finishers and related trades workers**	8 071	1 197 792	5.68 (5.44-5.93)	1.06 (1.04-1.08)	4 984	5.61 (5.46-5.76)
**Metal moulders, welders and sheet-metal workers**	3 041	476 331	5.64 (4.99-6.29)	1.09 (1.05-1.13)	1 412	5.25 (4.99-5.52)
**Machinery mechanics and fitters**	4 372	700 580	5.60 (4.95-6.24)	1.02 (0.99-1.06)	2 392	5.31 (5.11-5.52)
**Other personal services workers**	766	188 773	5.58 (5.12-6.04)	0.91 (0.85-0.98)	598	2.71 (2.50-2.92)
**Electrical and electronic equipment mechanics and fitters**	2 955	510 981	5.57 (5.24-5.91)	1.00 (0.96-1.04)	1 317	5.41 (5.13-5.69)
**Other office clerks**	8 224	1 697 405	5.55 (5.40-5.70)	1.04 (1.02-1.06)	3 816	4.53 (4.39-4.67)
**Mail carriers and sorting clerks**	1 529	272 771	5.54 (5.25-5.82)	1.05 (0.99-1.10)	808	5.19 (4.84-5.53)
**Library and filing clerks**	529	100 008	5.51 (4.94-6.09)	0.99 (0.90-1.07)	248	5.51 (4.85-6.18)
**Agricultural, fishery and related labourers**	259	46 526	5.50 (4.79-6.21)	1.08 (0.96-1.22)	158	5.97 (5.07-6.87)
**Retail salespersons**	8 216	1 972 898	5.47 (5.34-5.60)	1.03 (1.01-1.05)	4 610	3.79 (3.68-3.90)
**Forestry and related workers**	687	88 341	5.30 (4.61-5.99)	0.99 (0.91-1.06)	628	6.92 (6.40-7.44)
**Garment and related trades workers**	321	61 694	5.24 (4.60-5.87)	0.99 (0.89-1.11)	173	4.22 (3.61-4.84)
**Power-production and related plant operators**	714	108 900	5.24 (4.30-6.18)	0.98 (0.91-1.06)	611	5.78 (5.33-6.22)
**Painters, building structure cleaners and related trades workers**	1 653	275 391	5.20 (4.63-5.78)	1.05 (1.00-1.10)	971	4.62 (4.33-4.90)
**Total working population**	201 717	38 838 616	5.19 (5.17-5.22)	1.00 (1.00-1.00)	150 131	4.19 (4.17-4.21)
**Craft printing and related trades workers**	636	110 848	5.18 (4.75-5.60)	1.03 (0.95-1.12)	227	5.41 (4.73-6.10)
**Potters, glass-makers and related trades workers**	152	32 470	5.12 (4.27-5.96)	0.96 (0.82-1.13)	75	5.32 (4.15-6.50)
**Locomotive-engine drivers and related worker**	440	63 019	5.08 (4.18-5.98)	1.05 (0.96-1.16)	266	5.33 (4.71-5.95)
**Pelt, leather and shoemaking trades workers**	72	10 243	5.07 (3.79-6.36)	1.18 (0.92-1.48)	77	9.39 (7.39-11.39)
**Ships' deck crews and related workers**	178	23 634	5.03 (2.31-7.74)	1.14 (0.98-1.32)	113	6.59 (5.42-7.77)
**Office secretaries and data entry operators**	2 799	726 622	4.99 (4.56-5.42)	0.87 (0.83-0.90)	1 207	3.67 (3.46-3.87)

**Market gardeners and crop growers**	2 168	326 780	4.94 (4.67-5.20)	0.99 (0.95-1.03)	1 404	5.36 (5.09-5.63)
**Administrative professionals of special-interest organisations**	671	122 718	4.93 (4.54-5.31)	0.96 (0.89-1.04)	406	5.56 (5.03-6.09)
**Numerical clerks**	4 114	1 008 605	4.92 (4.67-5.16)	0.92 (0.89-0.95)	1 869	3.65 (3.48-3.81)
**Business services agents and trade brokers**	1 320	291 633	4.90 (4.63-5.17)	0.94 (0.89-0.99)	807	3.76 (3.50-4.01)
**Religious associate professionals**	104	19 940	4.89 (3.85-5.93)	0.87 (0.71-1.05)	67	5.05 (3.87-6.23)
**Crop and animal producers**	1 488	191 067	4.85 (4.48-5.22)	0.95 (0.90-1.00)	793	5.61 (5.23-5.99)
**Nursing associate professionals**	2 521	818 442	4.77 (4.38-5.17)	0.79 (0.76-0.82)	1 596	2.68 (2.55-2.81)
**Other teaching associate professionals**	670	131 851	4.77 (4.41-5.13)	0.93 (0.86-1.00)	362	4.95 (4.45-5.45)
**Managers of small enterprises**	7 382	1 352 144	4.67 (4.55-4.79)	0.92 (0.90-0.94)	3 445	4.39 (4.25-4.53)
**Customs, tax and related government associate professionals**	1 261	288 657	4.58 (4.26-4.90)	0.90 (0.85-0.95)	794	3.88 (3.61-4.14)
**Animal producers and related workers**	1 445	254 436	4.53 (4.22-4.84)	0.84 (0.80-0.88)	650	3.64 (3.37-3.92)
**Computer associate professionals**	2 090	584 301	4.53 (4.27-4.79)	0.88 (0.85-0.92)	1 106	3.28 (3.09-3.47)
**Precision workers in metal and related materials**	397	71 093	4.50 (3.89-5.11)	0.90 (0.82-1.00)	236	4.75 (4.15-5.34)
**Senior officials of special-interest organisations**	124	20 327	4.50 (3.59-5.41)	0.89 (0.74-1.06)	64	5.46 (4.16-6.76)
**Health professionals (except nursing)**	2 309	510 394	4.39 (4.20-4.57)	0.84 (0.81-0.88)	1 582	3.71 (3.53-3.89)
**Pre-primary education teaching associate professionals**	2 430	931 455	4.30 (3.90-4.70)	0.72 (0.69-0.75)	1 696	2.24 (2.13-2.34)
**Accountants**	4 993	1 356 161	4.29 (4.16-4.43)	0.80 (0.78-0.82)	2 436	3.23 (3.10-3.35)
**Ship and aircraft controllers and technicians**	490	92 485	4.26 (3.01-5.50)	0.82 (0.75-0.90)	261	3.92 (3.46-4.39)
**Handicraft workers in wood, textile, leather and related materials**	56	8 460	4.25 (2.78-5.72)	0.89 (0.67-1.16)	33	7.55 (5.07-10.03)
**Finance and sales associate professionals**	10 306	2 597 942	4.16 (4.08-4.25)	0.80 (0.79-0.82)	5 391	3.17 (3.09-3.26)
**Engineers and technicians**	9 752	2 064 837	4.12 (4.00-4.25)	0.80 (0.79-0.82)	4 422	3.88 (3.77-3.99)
**Optical and electronic equipment operators**	553	134 137	4.11 (3.70-4.52)	0.78 (0.72-0.85)	349	3.38 (3.03-3.73)
**Psychologists, social work and related professionals**	1 486	428 609	4.08 (3.83-4.33)	0.77 (0.73-0.81)	920	2.90 (2.71-3.08)
**Public service administrative professionals**	3 643	841 592	4.08 (3.94-4.21)	0.79 (0.76-0.82)	1 657	3.23 (3.08-3.38)
**Nursing and midwifery professionals**	1 545	516 842	4.02 (3.56-4.49)	0.69 (0.65-0.72)	690	2.22 (2.06-2.38)
**Life science technicians**	440	131 035	3.96 (3.32-4.60)	0.76 (0.69-0.83)	252	2.80 (2.46-3.14)
**Safety and quality inspectors**	814	146 087	3.96 (3.58-4.34)	0.83 (0.78-0.89)	314	4.02 (3.59-4.46)
**Other teaching professionals**	1 104	297 704	3.92 (3.68-4.16)	0.74 (0.70-0.78)	660	2.94 (2.72-3.16)
**Police officers and detectives**	906	180 335	3.91 (3.46-4.37)	0.82 (0.77-0.88)	343	2.85 (2.55-3.14)
**Elementary school teachers**	3 613	1 143 754	3.90 (3.76-4.05)	0.71 (0.68-0.73)	1 841	2.50 (2.39-2.62)
**Wood treaters, cabinet-makers and related trades workers**	192	40 389	3.86 (3.08-4.65)	0.77 (0.67-0.89)	97	4.31 (3.47-5.15)
**Production and operations managers**	5 019	1 164 398	3.81 (3.70-3.93)	0.75 (0.73-0.77)	2 370	2.82 (2.71-2.94)
**Directors and chief executives**	2 094	395 930	3.69 (3.42-3.96)	0.77 (0.74-0.80)	978	3.67 (3.44-3.89)

**Computer scientists**	3 256	1 171 927	3.68 (3.49-3.87)	0.67 (0.65-0.69)	1 900	2.22 (2.12-2.32)
**Artistic, entertainment and sports associate professionals**	559	219 412	3.67 (3.33-4.01)	0.67 (0.61-0.73)	308	2.09 (1.86-2.32)
**Senior high school teachers**	3 001	809 518	3.49 (3.37-3.62)	0.67 (0.65-0.70)	1 630	3.06 (2.91-3.21)
**Legal professionals**	688	201 168	3.47 (3.20-3.74)	0.67 (0.62-0.72)	375	2.51 (2.26-2.76)
**Agronomy and forestry technicians**	275	60 351	3.45 (2.90-4.00)	0.72 (0.64-0.81)	112	3.70 (3.02-4.37)
**Special education teaching professionals**	724	212 691	3.45 (3.11-3.79)	0.61 (0.56-0.65)	220	2.72 (2.37-3.08)
**Business professionals**	5 064	1 627 884	3.41 (3.32-3.51)	0.64 (0.63-0.66)	2 442	2.47 (2.38-2.57)
**Writers and creative or performing artists**	1 521	548 912	3.38 (3.21-3.55)	0.63 (0.60-0.66)	848	2.20 (2.05-2.35)
**Armed forces**	765	149 740	3.33 (2.30-4.36)	0.83 (0.77-0.89)	342	3.80 (3.40-4.19)
**Other specialist managers**	4 303	1 232 885	3.28 (3.16-3.40)	0.63 (0.61-0.65)	1 636	2.29 (2.18-2.40)
**Archivists, librarians and related information professionals**	418	132 286	3.25 (2.91-3.59)	0.63 (0.57-0.69)	207	2.36 (2.04-2.68)
**Health associate professionals (except nursing)**	1 022	425 469	3.23 (2.97-3.49)	0.57 (0.54-0.61)	607	1.83 (1.69-1.98)
**Social science and linguistics professionals (except social work professionals)**	282	102 806	3.17 (2.79-3.54)	0.60 (0.53-0.67)	150	2.66 (2.24-3.08)
**Legislators and senior government officials**	174	36 797	3.10 (2.55-3.64)	0.63 (0.54-0.74)	110	3.03 (2.47-3.58)
**Architects, engineers and related professionals**	3 040	1 053 591	2.85 (2.69-3.00)	0.57 (0.55-0.59)	1 643	2.40 (2.29-2.52)
**Life science professionals**	165	71 450	2.84 (2.32-3.36)	0.50 (0.42-0.58)	73	1.87 (1.44-2.29)
**College and university teachers**	1 142	468 057	2.74 (2.58-2.91)	0.53 (0.50-0.56)	583	2.11 (1.94-2.28)
**Physicists, chemists and related professionals**	272	120 565	2.68 (2.29-3.07)	0.48 (0.43-0.54)	103	1.70 (1.38-2.03)
**Mathematicians and statisticians**	58	33 490	1.70 (1.22-2.18)	0.35 (0.27-0.45)	20	1.38 (0.78-1.99)
**Fashion and other models**	1	1 686	1.30 (0.00-3.84)	0.18 (0.00-1.01)	1	1.30 (0.00-3.83)

ESM Table 6. Prevalence of overweight and physical fitness (W_{max}) in men born 1937-79. Data from the military conscription registry.

Occupation	No.	% BMI ≥25	Median W_{max}
Manufacturing labourers[1]	25 651	14.1%	247
Motor-vehicle drivers[1]	81 858	16.3%	240
Agricultural and other mobile plant operators[1]	36 586	16.8%	247
Personal carers[1b]	58 550	12.7%	260
Stores and transport clerks	62 221	13.4%	256
Machine operators[1]	26 354	13.6%	256
Metal- and mineral-products machine operators[1]	46 141	14.4%	252
Office clerks	30 820	10.3%	258
Retail salespersons[1]	74 024	11.7%	268
Metal moulders, welders and sheet-metal workers[1]	36 546	13.5%	250
Assemblers[1]	48 203	13.6%	259
Building finishers and related trades workers[1]	89 149	11.3%	247
Machinery mechanics and fitters[1]	56 977	13.6%	245
Total working population	1 312 959	10.7%	254
Electrical and electronic equipment mechanics[1]	38 267	10.7%	255
Managers of small enterprises	75 910	9.0%	252
Construction workers[1]	91 565	12.2%	255
Computer technicians and data operators	46 481	10.2%	270
Accountants	26 856	8.1%	257
Finance and sales associate professionals	142 073	8.5%	268
Elementary school teachers	20 995	8.9%	273
Engineers and technicians	139 895	9.0%	263
Public service administrative professionals	25 918	8.2%	251
Directors and chief executives	26 689	7.3%	251
Production and operations managers	63 877	8.8%	257
Computer scientists	89 056	8.0%	278
Senior high school teachers	26 614	8.3%	255
Specialist managers	75 122	7.0%	264
Business professionals	67 382	6.2%	268
Architects and civil engineers	75 170	6.8%	276
College and university teachers	18 393	6.5%	276

[1] Occupations classified as being in the lowest socio-economic status group. i.e. "skilled and unskilled manual workers" according to Statistics Sweden. [b]Personal carers includes child-care workers, assistant nurses, hospital ward assistants, home-based personal care, attendants in psychiatric care and dental nurses.

ESM Table 7. Prevalence of overweight and smoking in the first trimester in women born 1937-79. Data from the medical birth registry.

Occupation	No.	% BMI ≥25	% Smokers
Manufacturing labourers[1]	13 609	29.3%	29.8%
Cleaners[1]	49 849	29.3%	28.6%
Kitchen assistants[1]	42 356	29.9%	24.0%
Cooks, waitresses and housekeepers[1]	31 390	28.5%	22.8%
Personal carers[1b]	327 744	28.1%	23.5%
Cashiers, tellers and related clerks[1]	18 754	28.2%	18.2%
Social work associate professionals[1]	26 374	26.3%	19.5%
Office clerks	84 281	22.9%	17.3%
Client information clerks	40 262	26.7%	15.5%
Total working population	1 139 788	23.9%	16.7%
Retail salespersons[1]	97 774	26.4%	18.1%
Numerical clerks	56 896	22.0%	16.4%
Office secretaries and data entry operators	37 142	21.2%	15.8%
Health professionals (except nursing)[c]	18 586	14.9%	3.1%
Managers of small enterprises	31 760	21.4%	15.7%
Engineers and technicians	27 440	22.7%	8.5%
Accountants	72 067	20.5%	12.8%
Nurses	54 416	21.6%	10.8%
Public service administrative professionals	35 527	20.1%	10.8%
Psychologists and social workers	23 950	21.3%	12.8%
Finance and sales associate professionals	77 896	21.0%	11.1%
Preschool and recreation teachers	67 762	23.6%	9.0%
Computer scientists	24 850	21.3%	7.2%
Production and operations managers	37 814	20.6%	12.7%
Midwifes and specialist nurses	31 828	18.5%	9.7%
Business professionals	68 119	18.0%	8.1%
Elementary school teachers	63 532	22.4%	7.8%
Senior high school teachers	30 105	21.2%	8.6%
Specialist managers	29 997	17.5%	8.3%
Writers, creative or performing artists	23 207	17.8%	6.5%
Physiotherapists and dental hygienists	25 464	17.9%	5.7%

[1] Occupations classified as being in the lowest socio-economic status group. i.e. "skilled and unskilled manual workers" according to Statistics Sweden. [b]Personal carers includes child-care workers, assistant nurses, hospital ward assistants, home-based personal care, attendants in psychiatric care and dental nurses .[c] Health professionals includes physicians, veterinarians, dentists, pharmacists and speech therapists.

ESM Figure 1. Register linkage

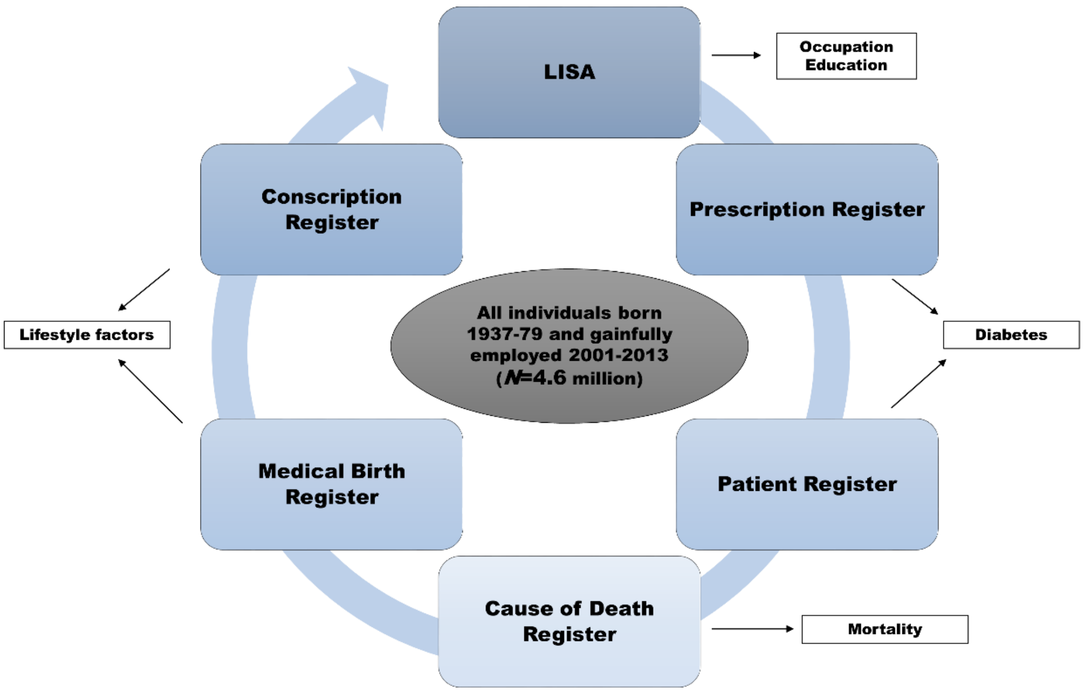

Supplement: Supplementary file 1 — (PDF 607 kb) [file 125_2019_4997_MOESM1_ESM.pdf]
